# Li-rich Thin Film Cathode Prepared by Pulsed Laser Deposition

**DOI:** 10.1038/srep03332

**Published:** 2013-11-26

**Authors:** Binggong Yan, Jichang Liu, Bohang Song, Pengfei Xiao, Li Lu

**Affiliations:** 1 State Key Laboratory of Advanced Design and Manufacturing for Vehicle Body, Hunan University, Changsha, P.R. China 410082; 2 Department of Mechanical Engineering National University of Singapore, Singapore 117576

## Abstract

Li-rich layer-structured cathode thin films are prepared by pulsed laser deposition. X-ray diffraction (XRD), field emission scanning electron microscope (FESEM), X-ray photoelectron spectroscopy (XPS) and electrochemical testing in half battery cells are used to characterize crystal structure, surface morphology, chemical valence states and electrochemical performance of these thin films, respectively. It is observed that partial layer to spinel transformation takes place during post annealing, and the layered structure further gradually transforms to spinel during electrochemical cycling based on the analysis of dQ/dV. Electrochemical measurement shows that the thin film electrode deposited at 350 mTorr and post-annealed at 800°C possesses the best performance.

Thin film lithium-ion batteries have received a lot of interests owing to their potential applications as micro-power sources for micro-devices such as smart cards, micro-sensors and implantable medical devices^1^,^2^. Since many thin film micro-batteries adopt metal lithium as an anode, development of the cathodes with high energy density becomes important. Many types of cathode thin film electrodes have been developed in the past decades including layer-structured LiCoO_2_, LiNi_x_Mn_y_Co_z_O_2_, spinel-structured LiNi_0.5_Mn_1.5_O_4_, LiMn_2_O_4_ and olivine-structured LiFePO_4_. Although reasonably high capacity and good cyclability have been achieved, the nature of low capacity of all the above cathode materials of about 140 ~ 160 mAh/g limits energy density for thin film micro-batteries.

In recent years, Li_2_MnO_3_-based Li-rich layered cathode materials, often written as xLi[Li_1/3_Mn_2/3_]O_2_·(1 − x) LiMO_2_ (M refers to Ni, Co or Mn), have attracted a lot of interests due to their high discharge capacity of about 250 mAh/g in a voltage range from 2.0 to 4.8 V vs. Li/Li^+^ 3,4, and showed good thermal and chemical stability at the same time, which makes it promised to be the next generation of cathode materials^5^. Since energy of the thin film micro-battery is extremely small due to the nature of thin electrode, development of high capacity cathode becomes important. Therefore in the present study, we intend to develop thin film electrode using the Li-rich layer-structured cathode material via pulsed laser deposition (PLD). Based on the study on bulk battery^3^,^4^, we identify Li_1.2_Mn_0.54_Ni_0.13_Co_0.13_O_2_ (or written as 0.55Li_2_MnO_3_·0.45Li[Mn_1/3_Ni_1/3_Co_1/3_]O_2_ based on mass ratio) as the target material. To avoid inter diffusion between current collector and the electrode, Au is used as the substrate^6^.

## Results

**Figure 1:**
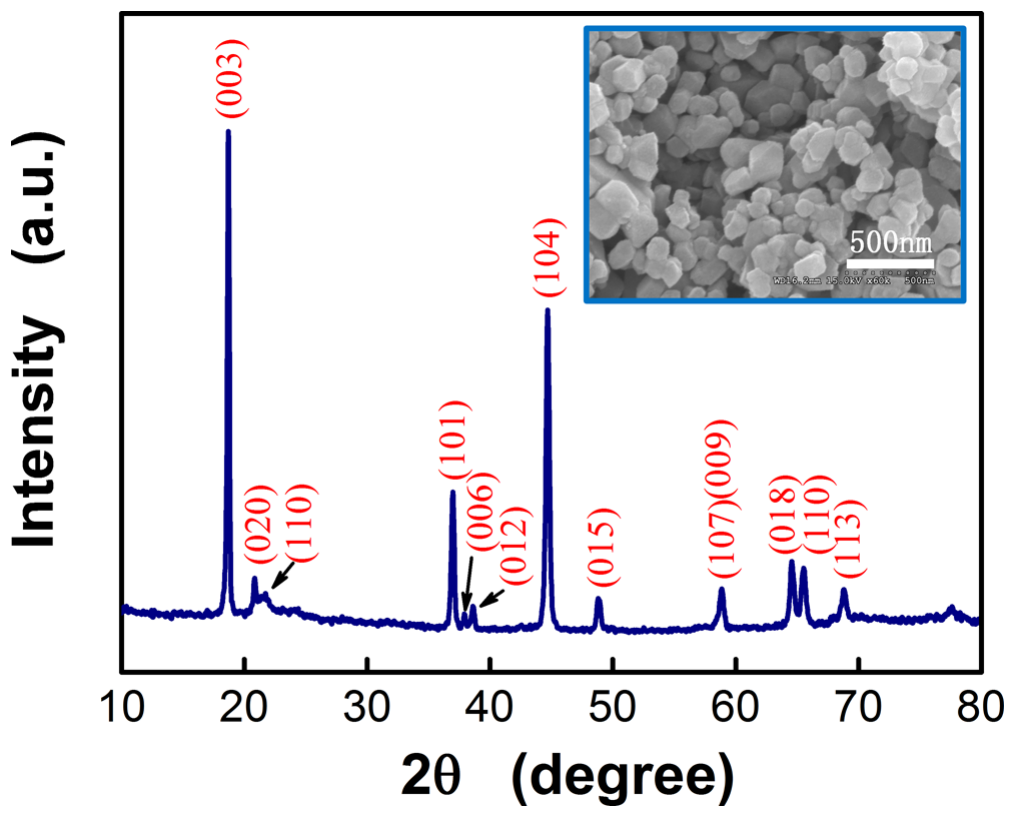
XRD spectra of the target material.

Fig. 1 shows the XRD spectrum of the Li_1.2_Mn_0.54_Ni_0.13_Co_0.13_O_2_ target which reveals a typical O3 layered structure with weak super structure reflections observed at about 21° ((020) and (110)) associated with the ordering of Li ions in the transition metal layers. Such observation is often recognized as one of the characters of Li-rich materials. The intensity ratio of (003)/(104) is about 1.53 (>1.2) which indicates that the cation mixing is very low. The clear splitting between the (006)/(012) and the (108)/(110) peaks according to the XRD pattern indicates that the target material possesses a typical ordering of layered structure. The inset FESEM image shows the particle size is about 300 nm.

**Figure 2:**
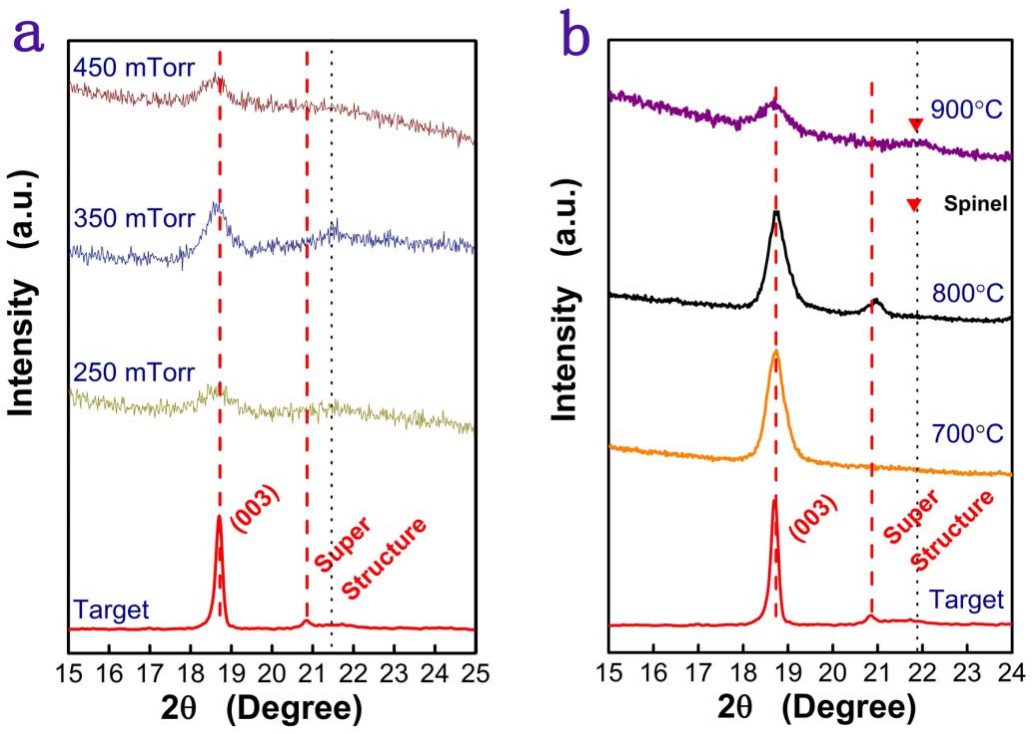
The XRD spectra of the as-deposited thin films grown at difference oxygen partial pressures (a), and those of the thin films that grew at 350 mTorr oxygen pressure, annealed at different temperatures.

Fig. 2(a) shows the XRD spectra of the thin films deposited at substrate temperature of 550°C and different oxygen partial pressures from 250 to 450 mTorr. As can be seen, the film that deposited at oxygen partial pressure 350 mTorr clearly shows a (003) peak and a super structure peak at about 21.4° that is often considered to be an evidence Li-rich layered structure^7^ whereas rest thin films deposited at lower or higher oxygen pressures do not clearly show the (003) diffraction. According to these observations, we can conclude that partial oxygen pressure of 350 mTorr is the suitable oxygen pressure for the growth of Li-rich thin film in the present condition. Although Li-rich layered structure has been obtained, the crystalinity of the as-deposited films was poor judged from the low diffraction intensity and broad diffraction peak. In order to improve crystallinity of the as-deposited thin films, the as-deposited thin films were post-annealed at different temperatures with pure oxygen flow.

Fig. 2(b) shows the XRD spectra of the films grown at 350 mTorr oxygen partial pressure after post annealing at different temperatures. As can be seen, 800°C annealed film shows clear (003) peak and clear super structure peak at 21° which are the characters of Li-rich materials^7^. Further increase in the annealing temperature to 900°C led to formation of a new phase identified by a new peak at about 22° accompanied by weak (003) from the layered structure. This new diffraction peak can be indexed to be the spinel^8^ which means that the structure of the film has changed after annealing at 900°C.

**Figure 3:**
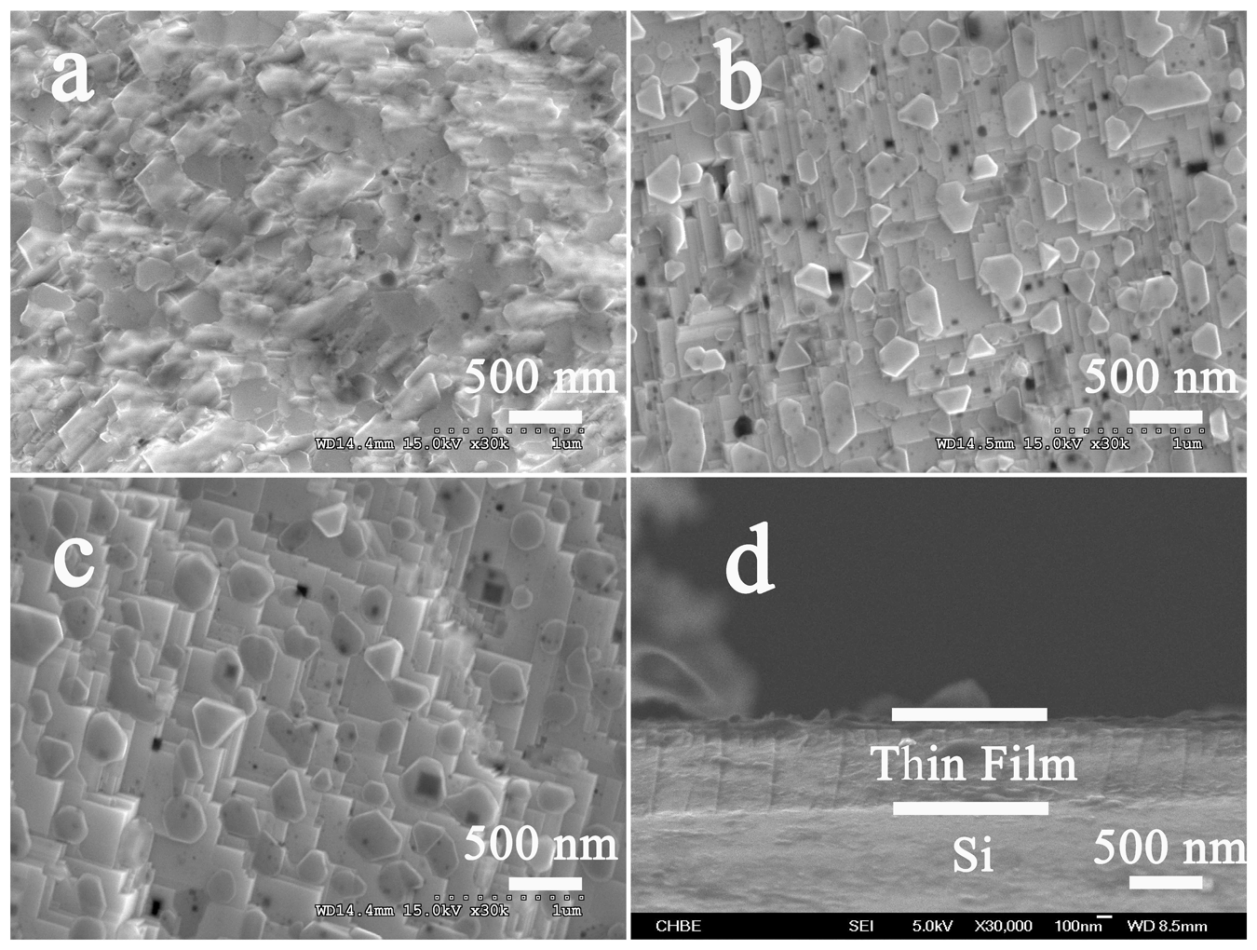
SEM images of the thin films post-annealed at different temperatures: (a) 700°C, (b) 800°C, (c) 900°C, (d) cross sectional image of as-deposited film.

Fig. 3 (a) through (c) respectively show the FESEM images of the thin films that grew at 350 mTorr oxygen partial pressure, and were post-annealed at different temperatures of 700°C, 800°C and 900°C. As can be seen, the film annealed at 700°C has relatively poor crystallinity while the films annealed at 800 and 900°C show better crystalline features. In addition, the particle size increases with the increasing annealing temperature as expected due to grain growth. Fig. 3 (d) shows the image of the cross section of the as-deposited film that prepared under 650°C and 350 mTorr oxygen pressure. The thickness of the film was estimated to be about 500 nm.

**Figure 4:**
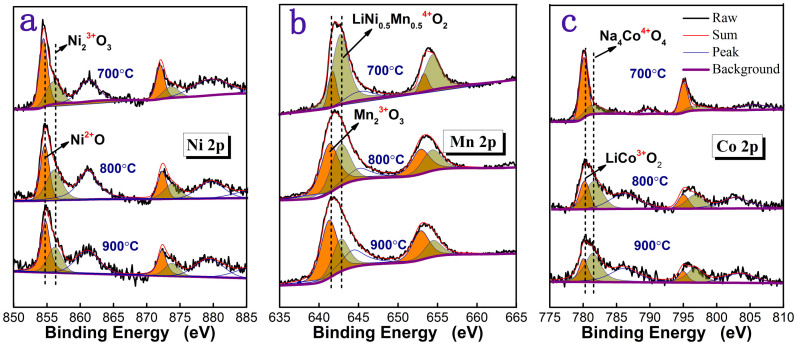
XPS spectra of Mn 2p, Ni 2p and Co 2p at different post annealing temperatures.

XPS spectra with fitted curves of the film deposited at 350 mTorr oxygen partial pressure and annealed from 700 to 900°C are shown in Fig. 4. According to Fig. 4 (a), Ni^2+^ 9 and Ni^3+^ 10 coexisted in the films while the area ratio remains almost the same after post annealed at different temperatures, which may imply that the post annealing process has very limited influence on Ni in the deposited films. Fig. 4 (b) reveals that the valences of Mn in the films annealed at three different temperatures possesses both 3+^11^ and 4+^12^, while the ratio of Mn^3+^ increases with increased annealing temperature. It is well known that Mn^3+^ is regularly associated with the formation of spinel-like structures^3^,^13^,^14^, and the increase of Mn^3+^ may imply growth of spinel-like structure during post annealing process. In fact, such observation is consistent with the results that reported by Koga^8^. Fig. 4 (c) shows the valence states of Co revealing both 3+^15^ and 4+^16^ and the area ratio of Co^4+^/Co^3+^ increases as the increase of annealing temperature which may be caused by the compensation as a result of valence decrease of Mn.

**Figure 5:**
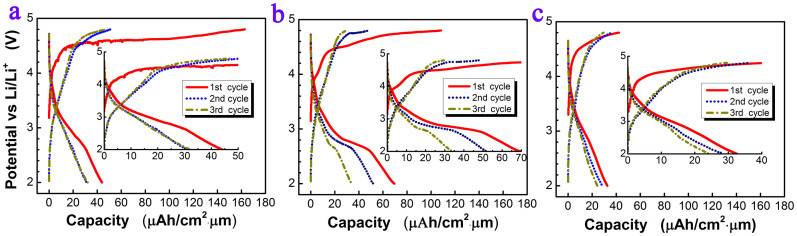
Charge-discharge curves of the thin films that annealed at different temperatures (a) 700°C, (b) 800°C, (c) 900°C.

Fig. 5 shows the charge-discharge curves of the first 3 cycles of the films that deposited at 350 mTorr oxygen partial pressure and post-annealed at three different temperatures. The film annealed at 700°C shows a long initial charge plateau at about 4.6 V while there is no obvious discharge plateau with relative low capacity which should be associated with its poor crystallization, whereas the film annealed at 800°C clearly shows three redox reactions during charge located at different voltages, first one from 3.9 V, another from 4.5 V and the highest one at about 4.7 V. The plateau at about 4.7 V is shorter than 700°C which may be caused by the Li loss at higher annealing temperature^17^. It is noted that a clear discharge plateau at about 2.8 V in the first discharge curve of the film annealed at 800°C, but it deteriorates very fast in the following cycles. The film annealed at 900°C shows the worst performance. No obvious charge plateau at 4.7 V in the initial charging process was observed, which could be imputed to the sever Li loss as a result of higher temperature.

**Figure 6:**
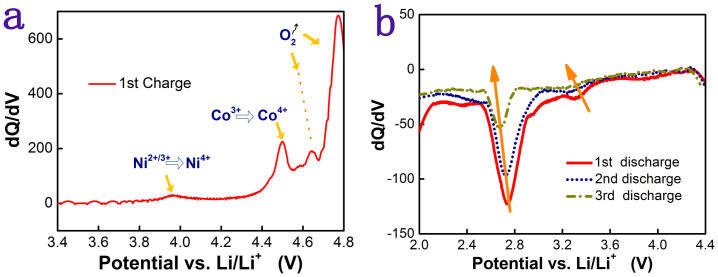
dQ/dV plots of (a) first charge process, (b) first 3 discharge processes based on charge-discharge of the film that annealed at 800°C.

**Figure 7:**
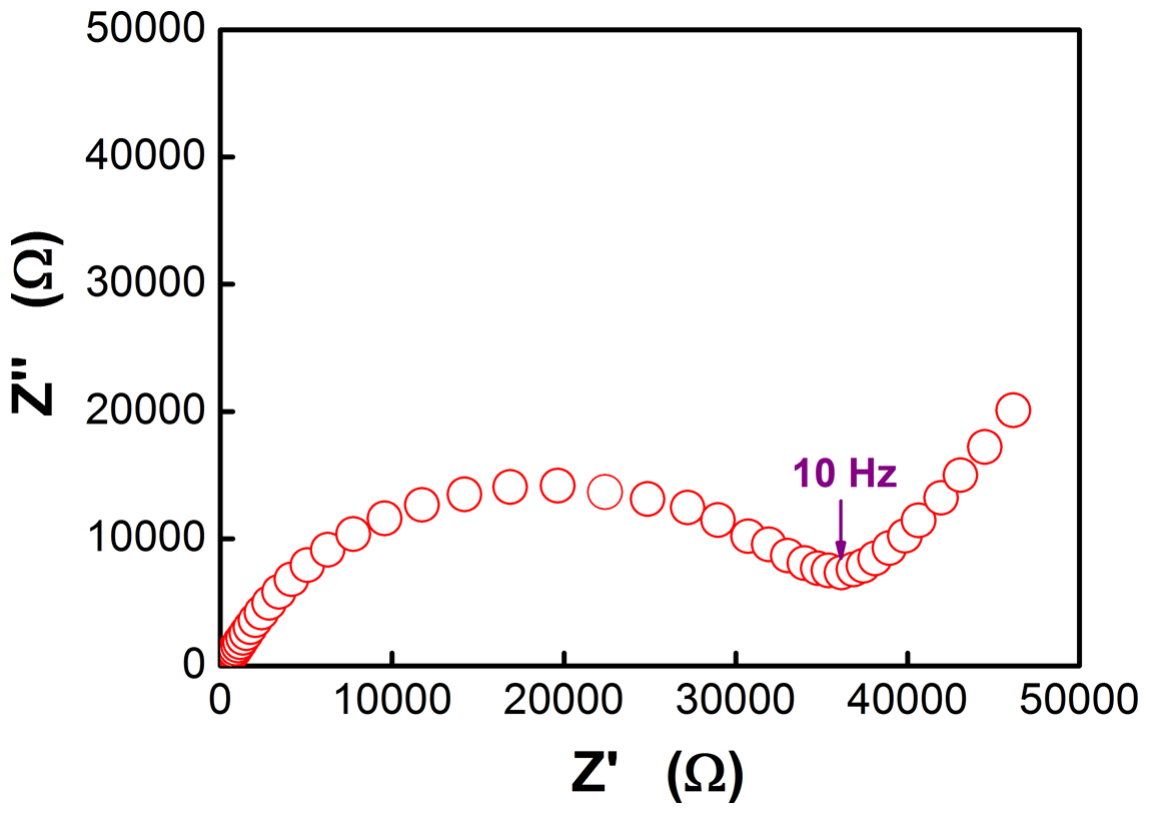
EIS spectrum of the battery based on the thin film cathode that annealed at 800°C.

Fig. 6 (a) shows the dQ/dV plot of the first charge process of the film that was deposited at 350 mTorr oxygen pressure and annealed at 800°C. As can be seen, Ni^2+^ was firstly oxidized to a higher oxidation state, the oxidization of Co^3+^ is located at about 4.5 V and the strongest peak located at higher than 4.6 V is ascribed to activation process of Li_2_MnO_3_. As it is know, Li_2_MnO_3_ in Li-rich material is normally activated electrochemically at about 4.4 V and redox potential of Co^+3^/Co^+4^ is lower than 4.4 V, the higher potential we obtained should result from the polarization which is associated with high resistance (37000 Ω) that can be identified in Fig. 7. Fig. 6 (b) shows the dQ/dV plots of the first three discharge processes. All of them exhibit two main peaks in the range of 2.0–4.8 V, one is located at about 2.8 V and another is located at about 3.4 V. The 3.4 V peaks should be associated with redox reaction in layered structure while the 2.8 V peaks should be correlated with Mn redox in other spinel-like structures. As this observation is abnormal in bulk Li-rich material, we suggest that the Li-rich layered structure as a result of first activated Li_2_MnO_3_ might be quickly deteriorated to spinel-like domained structures during the first charge-discharge process. Simonin, Atsushi etc. have verified this phase transformation using different methods in bulk Li-rich materials^3^,^4^,^18^,^19^. Since the crystalline and the atomic ordering may not be good enough in thin films, such phase transformation in thin films could be faster than bulk Li-rich counterpart while the resultant defected-spinels also deteriorate quickly.

## Discussion

Li-rich thin film cathode has been deposited on Au substrates using PLD. 350 mTorr oxygen partial pressure with 800°C post annealing temperature have been recognized as the best parameters for the growth of Li-rich thin film which shows the first discharge capacity as high as 70 μAh/cm^2^μm. Spinel phase partly formed during the post annealing process according to XPS results. Further transformation from the layered structure to spinel one during electrochemical cycling was also observed, which can be hypothesized to be caused by high in-plane constrain leading to a phase transformation.

**Figure 8:**
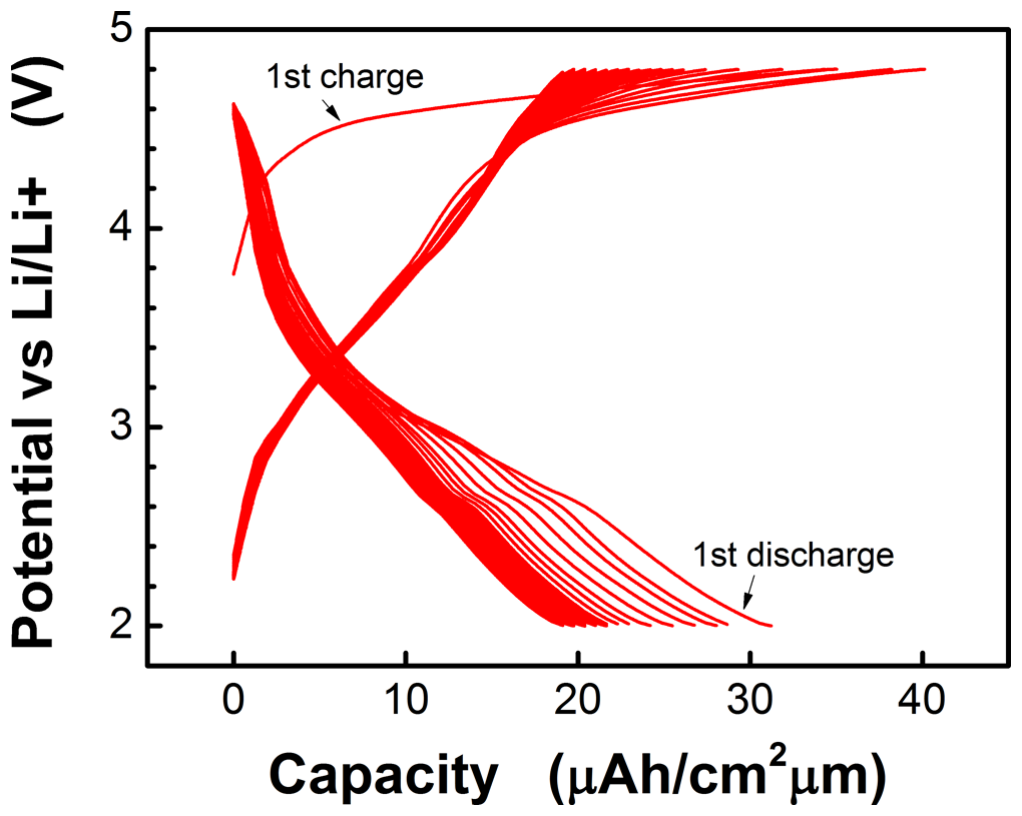
Charge-discharge curves of the thinner film that deposited under 350 mTorr and annealed at 800°C.

In order to verify the influence of thickness, we deposited thinner film (ca. 200 nm) using the optimized process parameters of 350 mTorr oxygen pressure and 800°C annealing temperature. Fig. 8 shows the charge-discharge curves of first 50 cycles of the thinner film. As can be seen, the first charge process performed similarly to typical Li-rich material and an obvious discharge process can be identified from 3.4 V to 3 V which should be owned to Li-rich layered structure. Since same testing current density was used, the charge-discharge rate of thinner film is better than that of the thick films. The capacity loss in the first 8 cycles is relatively large, which is mainly caused by layer to spinel transformation evidenced by the presence of discharge plateau that is located at about 2.6 V. We speculate that the layer to spinel transformation may be associated with two parameters, one of which is oxygen vacancies formed during deposition as well as post annealing. One top of that it might also be associated in-plane strain of the thin film that can assist transformation. After 8 charge/discharge cycles, the capacity loss became very small.

## Methods

The target material of composition Li_1.2_Mn_0.54_Ni_0.13_Co_0.13_O_2_ was made through conventional ball milling and solid solution process. The raw materials LiOH (Alfa, 99.9%), MnO_2_ (Alfa, 99.8%), Co (Alfa, 99.9%), Ni (Alfa, 99.9%) were used as the precursors. The molar ratio of Ni:Co:Mn was set to be 0.54:0.13:0.13 with 10% excess Li source to compensate the Li loss at high sintering temperature. The starting precursors were wet ball-milled for 2 hours in a steel container with two steel grinding balls. After drying in air at 80°C for 12 hours, the resultant mixture was cold pressed into a pellet of 26 mm in diameter and about 4 mm in thickness. The cold compact was sintered at 900°C for 24 hours to obtain a solid target. The structure of the target was verified using a powder X-ray diffractometer (Shimadzu 6000) with Cu Kα radiation (λ = 1.5418 Å).

A Lambda Physik KrF excimer laser beam (248 nm, 180 mJ) was used for thin film deposition at a repetition frequency of 10 Hz. Thin films were deposited on Au substrates at 650°C and different oxygen partial pressures. The target-substrate distance was kept at 20 mm. The as-deposited thin films were post-annealed at different post annealing temperatures of 700°C, 800°C and 900°C with oxygen flow for 40 min. X-Ray Photoelectron Spectroscopy (XPS) was used to identify the chemical valence states of transition metals, calibration was done by C1s (284.6 eV) spectrum of adventitious carbon that exists on all thin films. The structures of the as-deposited and annealed films were investigated by X-ray diffraction (XRD). Surface morphology of the thin films was characterized using a Hitachi S-4100 field emission scanning electron microscopy (FESEM). The thickness of thin films was estimated according to the cross-section image of FESEM of the sample which was deposited on the Si substrate.

Half thin film battery cells were assembled in an argon-filled glove box using the deposited Li-rich thin film as the working electrode, metal Li foil as the counter electrode and refernece electrode, two pieces of separators (Celgard 2500) and 7–10 drops of electrolyte (1 M LiPF6 in EC:DEC = 1:1 organic solutions). Swageloks were used for half-battery assembling. The charge-discharge tests were carried out using Neware Battery Test Station. All the tests were conducted at a constant current density of 2 μA/cm^2^ between 2.0 V and 4.8 V at room temperature. AC-impedance measurement was performed before charge-discharge in the frequency range from 1 MHz to 0.01 Hz using Solartron 1247.

## Author Contributions

L.L. and J.L. designed the experiments, B.Y. did the experiments and wrote the main manuscript text, B.S. and P.X. prepared figure 4 and figure 6. All authors discussed the results and reviewed the manuscript.
